# Editorial: Quality of care of glioma patients

**DOI:** 10.3389/fneur.2022.1041388

**Published:** 2022-10-06

**Authors:** Marie-Therese Forster, Philip De Witt Hamer, Shawn L. Hervey-Jumper, Mirjam Renovanz

**Affiliations:** ^1^Department of Neurosurgery, University Hospital Frankfurt, Frankfurt, Germany; ^2^Department of Neurosurgery, Neurosurgical Center Amsterdam, Amsterdam, Netherlands; ^3^Department of Neurological Surgery, University of California, San Francisco, San Francisco, CA, United States; ^4^Department of Neurology and Interdisciplinary Neuro-Oncology, Hertie Institute for Clinical Brain Research, Eberhard-Karls University of Tübingen, Tübingen, Germany; ^5^Center for Neuro-Oncology, Comprehensive Cancer Center Tübingen-Stuttgart, University Hospital of Tuebingen, Eberhard Karls University of Tübingen, Tübingen, Germany; ^6^Department of Neurosurgery, University Hospital Tübingen, Eberhard Karls University Tübingen, Tübingen, Germany

**Keywords:** quality of care, glioma, quality of life, neurocognition, new technologies

Over many decades, extent of tumor resection and overall survival were almost the sole parameters by which treatment success and quality of care of glioma patients had been measured. With the advent of new systemic therapy approaches and new imaging technologies, with the increasing understanding of brain connectivity and of molecular mechanisms of glioma disease, an individualized patient-centered therapy for glioma has evolved. Thus, today, many patients, especially patients with IDH-mutated gliomas with a more favorable prognosis, see themselves confronted with a chronic disease rather than with an end-of-life perspective immediately after tumor diagnosis.

As a consequence, quality of care of glioma patients has been brought into the focus, mainly encompassing high-quality and shared decision making (1), excellent performance, outcome measures and treatment accessibility (2). However, with regard to high-end techniques, complex quality and process management and the holistic approach toward patients considering their biopsychosocial situation, quality of care in this context still is defined to high-income countries. This applies even more for patients with brain tumors, for whom highest incidence rates have been found in countries with high sociodemographic index levels, reflecting the lack of accessibility of advanced and costly imaging technologies as well as advanced neurological and neurosurgical services in many areas of the world (3, 4). Moreover, due to population growth, aging as well as the environmental and socioeconomic situation of health care—including rising inflation and the shortage of qualified healthcare workers—the maintenance of quality of care of glioma patients will be challenging in the future.

Having all these healthcare system and healthcare service quality issues in mind, we aimed at gathering new insights into the current quality of care of glioma patients in this special Research Topic.

## Neurocognition and quality of life

A discussion on what quality of care means in lower-grade glioma patients has been provided by Taillandier et al., pointing out the importance of an “interactive patient-centered medicine.” In their opinion article they elaborate on the limitations of randomized-controlled trials in neuro-oncology and argue in favor of well-conducted observational studies in order to understand the long-term evolution of glioma, with respect to neurocognitive and health-related quality of life (QoL) parameters instead of solely molecular markers.

Neurocognition and QoL is the topic of six publications in this article collection. Van Kessel et al. investigated whether glioma patients' preoperative neurocognitive performance had an impact on survival. They found that memory function added prognostic value in high-grade glioma patients (additionally to established pre-selected predictors), but not in patients with low-grade glioma. Dufner et al. pointed out the importance of assessing patients' psychosocial burden and mood disturbances during adjuvant tumor treatment, given the relation of depression and chemotherapy-induced nausea and vomiting. Two publications focused on the neurocognitive consequences of awake glioma surgery. Staub-Bartelt et al. found that planned awake surgery had no negative impact on the prevalence of distress, anxiety or depression in glioma patients. Reitz et al. observed not only a high number of patients experiencing anxiety and depression prior to awake surgery, but also clear improvements in this and all other neurocognitive domains at long-term postoperative follow-up. Moreover, all patients in their cohort were seizure-free after awake surgery, albeit under anti-convulsive medication in 81.5% of patients. Robe et al. equally addressed postoperative outcome concerning seizure-freedom in patients with low-grade glioma, and the benefits of early surgery in these patients. Thus, patients undergoing early surgery for low-grade glioma significantly later lost their ability to work after tumor diagnosis than patients who underwent tumor resection at least 6 months after diagnosis.

## Neurological function and frailty assessments

However, patients' pre- and post-operative neurological function, and thus, quality of life, clearly depends on glioma localization and invasion of eloquent regions. Thus, Coburger et al. reported on 83 patients with lower grade glioma (WHO grade II and III, according to the WHO 2016 classification) situated in regions involving motor and/or language function and observed permanent new postoperative deficits in 38.6% of their patients 3 months after surgery. They found that permanent new neurological deficits after surgery significantly correlated with preoperative neurological impairment and complete tumor resection.

While these authors did not address overall survival of their patients, another publication within this Research Topic, provided by Kasper et al., focusing on glioblastoma patients, found a clear association between patients' neurological function after surgery and overall survival. Similarly, Krenzlin et al. reported on a significant correlation of patients' frailty, assessed by The Geriatric eight health status screening tool (G8) and Groningen Frailty Index (GFI), and overall survival.

However, although QoL has increasingly been put into the focus of clinical trials of glioma patients, clinical assessment practice of patient-reported outcomes (PROs) still remains very heterogenous, as shown by Weiss Lucas et al. Investigating the use of PRO and neurocognition assessment practices throughout departments of surgical neuro-oncology in Germany, they observed that only a small majority of departments performed patient-centered screenings outside of clinical trials. As a consequence, the authors recommended a minimum number of PRO and neurocognitive assessments for routine clinical practice in their publication.

## Quality indicators and adverse events

While quality of care ultimately aims at excellent patient outcomes, quality of care highly depends on process quality. Besides defining quality indicators for quality of care assessments (5), detecting and reporting on adverse events and complications are therefore essential for continuously improving processes, and thus, improving the quality of care. Vecchio et al. addressed the issue of adverse events following surgery in lower-grade gliomas, using the Landriel–Ibanez classification (LIC) and the Therapy-Disability-Neurology (TDN) score, reporting on postoperative complications in 47.6% of their patients. Katzendobler et al. equally focused on severe adverse events; however, after stereotactic biopsies in 617 glioma patients, they only identified severe postoperative complications including complications requiring an intervention in 1.2% of cases. Moreover, they succeeded to establish an integrated diagnosis by stereotactic biopsy in 96.4% of their patients.

## Novel prognostic biomarkers

Regarding diagnosis and the prognostic value of gene expression profiling, Liu R. et al. reported on Guanine nucleotide-binding protein subunit gamma 12 (GNG12) as a novel biomarker, with high levels of GNG 12 expression representing an independent risk factor for poor prognosis in patients with glioma, regardless of the presence of IDH mutations or 1p/19q co-deletions.

## Uncommon co-morbidities and tumor presentations in adults with gliomas

The remaining two articles in this Research Topic focus on clinical themes with a relatively rare incidence in adults with glioma. El Rahal et al. retrospectively analyzed 1,800 glioblastoma patients of whom 2.1% had been treated for hydrocephalus by ventricular shunting. They observed symptomatic improvement in 95% of patients after shunting and the necessity of shunt revisions in 26% of patients, and concluded that hydrocephalus treatment in glioblastoma patients “might maintain patients' eligibility for crucial oncological therapy as well as quality of life.”

Finally, Liu H. et al. provided an analysis of 1257 patients with optic pathway gliomas and revealed OS rates of 93% 10 years after tumor diagnosis with treatments including surgery, radiation and chemotherapy not resulting in better prognoses.

To summarize, this article collection includes remarkable publications on the quality of care of glioma patients and emphasizes again the multifaceted nature of the Research Topic. We thank all the authors, colleagues and the team of Frontiers who contributed to this work.

## Author contributions

M-TF: conceptualization. M-TF and MR: data interpretation and writing—original draft. M-TF, PDWH, SH-J, and MR: data acquisition and writing—review and editing. All authors contributed to the article and approved the submitted version.

## Conflict of interest

The authors declare that the research was conducted in the absence of any commercial or financial relationships that could be construed as a potential conflict of interest.

## Publisher's note

All claims expressed in this article are solely those of the authors and do not necessarily represent those of their affiliated organizations, or those of the publisher, the editors and the reviewers. Any product that may be evaluated in this article, or claim that may be made by its manufacturer, is not guaranteed or endorsed by the publisher.
