# Corrigendum: Editorial: Quality of care of glioma patients

**DOI:** 10.3389/fneur.2022.1084412

**Published:** 2022-12-01

**Authors:** Marie-Therese Forster, Philip De Witt Hamer, Shawn L. Hervey-Jumper, Mirjam Renovanz

**Affiliations:** ^1^Department of Neurosurgery, University Hospital Frankfurt, Frankfurt, Germany; ^2^Department of Neurosurgery, Neurosurgical Center Amsterdam, Amsterdam, Netherlands; ^3^Department of Neurological Surgery, University of California, San Francisco, San Francisco, CA, United States; ^4^Department of Neurology and Interdisciplinary Neuro-Oncology, Hertie Institute for Clinical Brain Research, Eberhard-Karls University of Tübingen, Tübingen, Germany; ^5^Center for Neuro-Oncology, Comprehensive Cancer Center Tübingen-Stuttgart, University Hospital of Tuebingen, Eberhard Karls University of Tübingen, Tübingen, Germany; ^6^Department of Neurosurgery, University Hospital Tübingen, Eberhard Karls University Tübingen, Tübingen, Germany

**Keywords:** quality of care, glioma, quality of life, neurocognition, new technologies

In the published article, there was an error regarding the affiliations for “Mirjam Renovanz.” Mirjam Renovanz's affiliations should be:

“^4^ Department of Neurology and Interdisciplinary Neuro-Oncology, Hertie Institute for Clinical Brain Research, Eberhard-Karls University of Tübingen, Tübingen, Germany

^5^ Center for Neuro-Oncology, Comprehensive Cancer Center Tübingen-Stuttgart, University Hospital of Tuebingen, Eberhard Karls University of Tübingen, Tübingen, Germany

^6^ Department of Neurosurgery, University Hospital Tübingen, Eberhard Karls University Tübingen, Tübingen, Germany”

The authors apologize for this error and state that this does not change the scientific conclusions of the article in any way. The original article has been updated.

## Publisher's note

All claims expressed in this article are solely those of the authors and do not necessarily represent those of their affiliated organizations, or those of the publisher, the editors and the reviewers. Any product that may be evaluated in this article, or claim that may be made by its manufacturer, is not guaranteed or endorsed by the publisher.

